# Virtual screening–molecular docking–activity evaluation of *Ailanthus altissima* (Mill.) swingle bark in the treatment of ulcerative colitis

**DOI:** 10.1186/s12906-023-03991-0

**Published:** 2023-06-15

**Authors:** Shan-bo Ma, Lun Liu, Xiang Li, Yan-hua Xie, Xiao-peng Shi, Si-wang Wang

**Affiliations:** 1 The College of Life Sciences, Northwest University, 229 Taibai Road, Xi’an, 710069 Shaanxi China; 2 Department of Pharmacy, Xijing Hospital, Air Force Medical University, Xi’an, 710032 China

**Keywords:** *Ailanthus altissima* (Mill.) Swingle Bark, Virtual screening, Molecular Docking, Ulcerative Colitis, Evaluation

## Abstract

**Background:**

The dried bark of *Ailanthus altissima* (Mill.) Swingle is widely used in traditional Chinese medicine for the treatment of ulcerative colitis. The objective of this study was to explore the therapeutic basis of the dried bark of *Ailanthus altissima* (Mill.) Swingle for the treatment of ulcerative colitis based on Virtual Screening–Molecular Docking–Activity Evaluation technology.

**Methods:**

By searching the Traditional Chinese Medicine Systems Pharmacology TCMSP Database and Analysis Platform, 89 compounds were obtained from the chemical components of the dried bark of *Ailanthus altissima* (Mill.) Swingle. Then, after preliminarily screening the compounds based on Lipinski’s rule of five and other relevant conditions, the AutoDock Vina molecular docking software was used to evaluate the affinity of the compounds to ulcerative colitis-related target proteins and their binding modes through use of the scoring function to identify the best candidate compounds. Further verification of the compound’s properties was achieved through in vitro experiments.

**Results:**

Twenty-two compounds obtained from the secondary screening were molecularly docked with ulcerative colitis-related target proteins (IL-1R, TLR, EGFR, TGFR, and Wnt) using AutoDock Vina. The free energies of the highest scoring compounds binding to the active cavity of human IL-1R, TLR, EGFR, TGFR, and Wnt proteins were − 8.7, − 8.0, − 9.2, − 7.7, and − 8.5 kcal/mol, respectively. The potential compounds, dehydrocrebanine, ailanthone, and kaempferol, were obtained through scoring function and docking mode analysis. Furthermore, the potential compound ailanthone (1, 3, and 10 µM) was found to have no significant effect on cell proliferation, though at 10 µM it reduced the level of pro-inflammatory factors caused by lipopolysaccharide.

**Conclusion:**

Among the active components of the dried bark of Ailanthus altissima (Mill.) Swingle, ailanthone plays a major role in its anti-inflammatory properties. The present study shows that ailanthone has advantages in cell proliferation and in inhibiting of inflammation, but further animal research is needed to confirm its pharmaceutical potential.

## Background

Ulcerative colitis (UC), a nonspecific inflammatory bowel disease with an unknown etiology [1], often occurs in the colon and rectum and is diagnosed by colonoscopy [2, 3]. Patients with recurrent and prolonged UC are prone to cancer [4, 5], and UC has been classified as a refractory disease by the World Health Organization (WHO) [6]. Western medicine does not effectively control UC flares, and cannot prevent its recurrence [7]. In contrast to the long treatment cycle [8], adverse drug effects [9], high treatment costs [10, 11], and other disadvantages of Western medicine, traditional Chinese Medicine (TCM) has significant effects in the clinical treatment of UC [12].

The bark of *Ailanthus altissima* (Mill.) Swingle (BAA), which is also known as chun root bark, citrus white bark, ailanthus bark, and kucha bark, is commonly used in TCM. It has a bitter, astringent, and cold taste [13]. BAA can clear heat, dry dampness, astringe the intestines, and stop bleeding, and it has been used to treat chronic dysentery, enteritis, diarrhea, gastric and duodenal ulcers, blood in the stool, nocturnal emission, leucorrhea, and other diseases [14, 15]. Modern medical research has found that BAA and its preparations have a good curative effect on UC [13, 16–18]. A 100%-pure decoction of BAA has been shown to have inhibitory effects on *Shigella flexneri, Shigella sohnii*, *Bacillus typhi*, and *Escherichia coli in vitro*, and *Staphylococcus aureus* is moderately sensitive to it [19, 20]. BAA extract can also reduce the activity of nitric oxide (NO) free radicals and nitric oxide synthase (NOS) [18, 21], which are inflammatory factors associated with UC [22].

Molecular docking technology docks known small-molecule active substances with related target proteins, and can elucidate the mechanism of action between active TCM ingredients and targets at the molecular level [23, 24]. At present, molecular docking technology has played an important role in elucidating the mechanism of action between active TCM ingredients and related target proteins in many diseases, such as cardiovascular disease [25] and cancer [26]. Research on the treatment of UC is in the initial stage, and the pharmacodynamic substances that are effective against UC have not yet been fully investigated [27]. The molecular docking virtual screening technology used to screen the pharmacodynamic substances (active component groups) of BAA that are effective in the treatment of UC is relatively simple and can obtain accurate results. Various signaling pathways that are closely related to UC, including TLR/IL-1R, EGFR, TGFR and Wnt/β-catenin. Research has found that the TLR/IL-1R signaling pathways play a crucial role in host defense and inflammation [28], the EGFR and Wnt signaling pathways are closely related to the repair of colonic mucosal injury [29, 30], TGFR can also significantly promote the apoptosis of T cells [31], thereby exacerbating the development of UC [32]. Therefore, TLR, IL-1R, EGFR, TGFR, and Wnt can serve as docking proteins.

The aim of this study was to identify all of the known chemical components of BAA using the Traditional Chinese Medicine Systems Pharmacology (TCMSP) Database and Analysis Platform, combined with the five principles of drug-like molecules (Lipinski rule) and standard conditions, such as oral bioavailability and drug similarity, to preliminarily screen for compounds that could be developed into therapeutics. We set human interleukin-1 receptor (IL-1R), toll-like receptor (TLR), epidermal growth factor receptor (EGFR), transforming growth factor receptor (TGFR), and Wnt proteins as the targets of UC, and AutoDock Vina molecular docking software was used to sort the bioactive compounds of BAA by scoring function. Additionally, the primary-screening compound group and key targets of disease pathogenesis were also explored. The affinity and binding mode of the proteins were then used to screen for active ingredients with potential therapeutic effectiveness against UC. In addition, potential compounds were further tested in vitro.

## Materials and methods

### Construction of ligand library (compound)

The TCMSP Database and Analysis Platform were used to establish a preliminary-screening compound group for TCM (https://old.tcmsp-e.com/tcmsp.php) [33]. The small-molecule data were downloaded in mol2 format from the TCMSP database according to the small molecule MOLID number. The small molecules were then imported into ChemBio3D Ultra 14.0 (Cambridgesoft, Cambridge, MA, USA) for energy minimization. The minimum RMS gradient was set at 0.001, and the small molecule was saved in mol2 format. The optimized small molecule was imported into AutoDockTools (Scripps Research Institute, La Jolla, CA, USA) for hydrogenation, and the charge was calculated and assigned, in addition to the rotatable bond being set. The file was saved in “pdbqt” format.

### Preparation of receptors (proteins)

UC-related target protein data came from Protein Data Bank (PDB, http://www.rcsb.org/) [34]. The most relevant UC target proteins, including human IL-1R, TLR, EGFR, TGFR, and Wnt, were set as the receptor proteins. High resolution was preferred, and the original ligand had high structural similarity to the active ingredient to be docked. Pymol (DeLano Scientific LLC, Palo Alto, CA, USA) was used to remove protein crystal water and original ligands. The protein structure was imported into AutoDockTools (Scripps Research Institute, La Jolla, CA, USA) for hydrogenation, and the charge was calculated and assigned. The type of atom was specified, and the file was saved in “pdbqt” format.

### Docking settings

AutoDock Vina [35] (Scripps Research Institute, La Jolla, CA, USA) was used for docking, and the EGFR [36](PDB ID: 7LGS) target-related parameters were set as follows: center_x = 29.720,center_y = − 19.100, center_z = 5.833, and size of the grid box = 60 × 60 × 60 (the spacing between each grid point was 0.375Å).The rest of the parameters were the default settings. IL-1R (PDB ID: 5R8J) target-related parameters were set as follows: center_x = 38.545, center_y = 13.206, center_z = 68.747, and size of the grid box = 114 × 94 × 110 (the spacing between each grid point was 0.375Å); the rest of the parameters were the default settings. TLR1 (PDB ID: 7NT7) target-related parameters were set as follows: center_x = 38.062, center_y = 15.372, center_z = − 6.727, and size of the grid box was set to 84 × 106 × 120 (the spacing between each grid point was 0.375 Å).The rest of the parameters were the default settings. The Wnt-8 (PDB ID: 4FOA) target-related parameters were set as follows: center_x = − 62.066, center_y = − 9.0, center_z = 9.934, and size of the grid box = 126 × 126 × 126 (the spacing between each grid point was 0.375Å); the rest of the parameters were the default settings. TGFR (PDB ID: 5Q1L) target-related parameters were set as follows: center_x = 4.108, center_y = 9.252, center_z = 7.215, and size of the grid box was set to 60 × 60 × 60 (the spacing between each grid point was 0.375Å).The rest of the parameters were the default settings.

### Molecular docking between chemical components and target proteins

The compounds with the highest docking score for each protein were selected to visualize the docking results using Discovery Studio (Accelrys Software Inc., San Diego, USA).

### CCK-8 cytotoxicity assay

Human normal colonic epithelial NCM460 cells and mouse RAW264.7 macrophages were provided by the Cell Bank of the Chinese Academy of Sciences (Shanghai, China). We took the cells in the logarithmic growth phase (NCM460; RAW264.7), counted them, pipetted 200 µL of cell suspension into a 96-well plate, and set the cell density to 5 × 10^3^ cells per well. We divided the samples into blank and control groups, which were both placed in an incubator for routine culture for 24 h. We accurately weighed the test drug ailanthone, used DMSO to prepare a mother solution with a concentration of 200 µM, and then sequentially diluted it with DMSO to concentrations of 1, 3, 10, 30, and 50 µM for administration. The next day, in both the blank group and the control group, the medium was replaced with a fresh culture medium. Specifically, in the administration group, a culture medium containing drugs (1, 3, 10, 30, and 50 µM) was used, while we added 1 µL of DMSO to the solvent control group; afterward they were placed in an incubator for routine culture. After 24 h, 10 µL of CCK-8 solution was added to each well, and the cells were placed in an incubator for further incubation for 2.5 h. We measured the absorbance value A of each well at a wavelength of 450 nm with an enzyme-linked immunosorbent assay, calculated the average value of the absorbance of each group, and calculated the inhibition rate according to the following formula: Inhibition rate% = 100% × [(A control group − A administration group)/(A control group − A blank group)]. The IC_50_ values of drugs on cells (NCM460; RAW264.7) were calculated with GraphPad Prism software.

### Determination of anti-inflammatory activity

According to the CCK-8 cytotoxicity test, the concentration of the drug whose toxicity to Raw264.7 cells was less than the IC_50_ value was selected for anti-inflammatory activity detection, and drug concentrations of 1, 3, and 10 µM were selected. Raw264.7 cells in the logarithmic growth phase were taken and trypsinized; the cells were collected and centrifuged at 1000 rpm/min; the supernatant was discarded, and the cells were resuspended in a fresh culture medium. We counted the cells, adjusted the cell density, pipetted 100 µL of the cell suspension into a 24-well plate, and then added 700 µL of the culture medium to achieve a cell density of 1 × 10^5^ cells per well. We set the blank group and the control group and placed them in an incubator for routine culture for 24 h. The test drug ailanthone was precisely weighed, and the mother solution was prepared with DMSO to achieve a concentration of 200 µM, which was sequentially diluted with DMSO to concentrations of 1, 3, and 10 µM for administration. The DMSO content of the administered cells was less than or equal to 1%. The cells were first incubated with drugs for 2 h, then exposed to LPS with a final concentration of 1 µg/mL, and finally co-cultured for 24 h. After 24 h, the cell supernatant of each experimental group was collected, and the absorbance value was detected by a microplate reader in accordance with the operation instructions of the ELISA kit. The contents of tumor necrosis factor-α (TNF-α), interleukin-6 (IL-6), and interleukin-1β (IL-1β) in the supernatant were determined.

### Statistical analysis

The statistical software used was GraphPad Prism 8 (La Jolla, CA, USA), and *P* < 0.05 indicated a statistically significant difference.

## Results

### Preliminary screening of compounds in BAA

Compounds were searched with BAA as the keyword, and 89 small-molecule chemical components were retrieved from the TCMSP database. Eighty-nine compound libraries were initially constructed. The five principles of drug-like and other related screening conditions were used for secondary screening: (1) molecular weight (MW) less than 500; (2) lipid–water partition coefficient (ALogP) less than 5; (3) number of hydrogen-bond donors (Hdon) less than 5; (4) number of hydrogen-bond receptors (Hacc) less than 10; (5) oral bioavailability (OB) greater than 50; and (6) drug similarity (DL) greater than 0.18. The secondary screening resulted in 22 compounds. As shown in Table 1, these 22 compounds were suggested to have good drug-like properties, and were saved in mol2 format for future use.

**Table 1:** Compounds that met the initial screening conditions

Mol ID	Molecule Name
MOL000422	Kaempferol
MOL006276	SMR000232320
MOL006277	Shinjudilactone
MOL006278	Shinjulactone A
MOL006279	Shinjulactone B
MOL006280	Shinjulactone C
MOL006281	Shinjulactone K
MOL006284	Ailanthone
MOL006285	Ailantinol A
MOL006294	2,3,5,6,9,11,12,15,16,1y-decahydro-IH-cyclopentalalphenanthren-iy-yl-methylheptane-2,3,4-triol
MOL006300	1-Hydroxy-canthin-6-one
MOL006301	1-Methoxycanthinone
MOL006302	2-Hydroxy-canthin-6-one
MOL006303	4,5-Dihydrocanthin-6-one
MOL006304	4-Hydroxy-canthin-6-one
MOL006306	5-Methoxycanthin-6-one
MOL006308	66762-19-4
MOL006309	Amarolide 11-acetate
MOL006310	Amarolide
MOL006311	Artelin
MOL006314	Canthin-6-one
MOL006315	Dehydrocrebanine

### Scoring function ranking of secondary-screening chemical components and target proteins

The 22 compounds obtained from the secondary screening were molecularly docked with human IL-1R, TLR, EGFR, TGFR, and Wnt proteins using AutoDock Vina software. The binding conformation was scored using a scoring function based on shape matching and energy matching. The docking results of the compounds and receptors were sorted in descending order of the total score, and the results are shown in Table 2. The free energies of binding to the active cavities of human IL-1R, TLR, EGFR, TGFR, and Wnt protein of the highest scoring compound obtained by AutoDock Vina software were − 8.7, − 8.0, − 9.2, − 7.7, and − 8.5 kcal/mol, respectively. A larger absolute value of the binding constant indicates that lower free energy is required for the compound to bind. It is generally believed that when the absolute value is greater than 7, the combination of the compound and the protein is more likely.

**Table 2:** Molecular docking function scoring and ranking results

EGFR	Affinity kcal/mol	IL-1R	Affinity kcal/mol	TGFR	Affinity kcal/mol	TLR1	Affinity kcal/mol	Wnt8	Affinity kcal/mol
MOL006315	−8.7	MOL006284	−8.0	MOL000422	−9.2	MOL006284	−7.7	MOL006284	−8.5
MOL006301	−8.5	MOL006310	−7.8	MOL006311	−8.9	MOL006277	−7.6	MOL006278	−8.4
MOL006277	−8.4	MOL006277	−7.7	MOL006315	−8.7	MOL006278	−7.6	MOL006279	−8.1
MOL006302	−8.4	MOL006285	−7.7	MOL006303	−8.6	MOL006300	−7.4	MOL006309	−8.0
MOL006308	−8.4	MOL006309	−7.6	MOL006304	−8.6	MOL006294	−7.2	MOL006310	−7.9
MOL000422	−8.3	MOL006278	−7.5	MOL006301	−8.5	MOL006303	−7.2	MOL006277	−7.7
MOL006304	−8.3	MOL006281	−7.1	MOL006302	−8.5	MOL006310	−7.1	MOL006294	−7.7
MOL006306	−8.2	MOL006315	−7.1	MOL006306	−8.5	MOL006280	−7.0	MOL006280	−7.3
MOL006311	−8.2	MOL000422	−6.9	MOL006284	−8.4	MOL006301	−6.9	MOL006281	−7.1
MOL006314	−8.2	MOL006311	−6.8	MOL006300	−8.4	MOL006306	−6.9	MOL006311	−7.1
MOL006300	−8.1	MOL006279	−6.7	MOL006314	−8.4	MOL006314	−6.9	MOL006315	−7.1
MOL006284	−8.0	MOL006280	−6.5	MOL006277	−8.2	MOL006285	−6.8	MOL000422	−7.0
MOL006303	−8.0	MOL006276	−6.3	MOL006308	−8.2	MOL006309	−6.8	MOL006285	−7.0
MOL006310	−8.0	MOL006294	−6.1	MOL006280	−7.7	MOL006315	−6.8	MOL006276	−6.8
MOL006309	−7.9	MOL006303	−6.0	MOL006310	−7.7	MOL006304	−6.7	MOL006302	−6.7
MOL006285	−7.8	MOL006304	−6.0	MOL006278	−7.4	MOL006279	−6.6	MOL006301	−6.4
MOL006279	−7.7	MOL006300	−5.9	MOL006279	−7.4	MOL006311	−6.6	MOL006306	−6.4
MOL006276	−7.6	MOL006302	−5.8	MOL006294	−7.3	MOL000422	−6.5	MOL006300	−6.3
MOL006280	−7.6	MOL006306	−5.8	MOL006276	−7.2	MOL006281	−6.3	MOL006303	−6.2
MOL006294	−7.6	MOL006308	−5.8	MOL006309	−7.2	MOL006276	−6.1	MOL006304	−6.2
MOL006278	−7.4	MOL006314	−5.8	MOL006285	−7.1	MOL006308	−6.0	MOL006314	−6.2
MOL006281	−6.5	MOL006301	−5.7	MOL006281	−7.0	MOL006302	−5.9	MOL006308	−6.1

### Binding pattern of the highest scoring compound and target protein

**Fig. 1:**
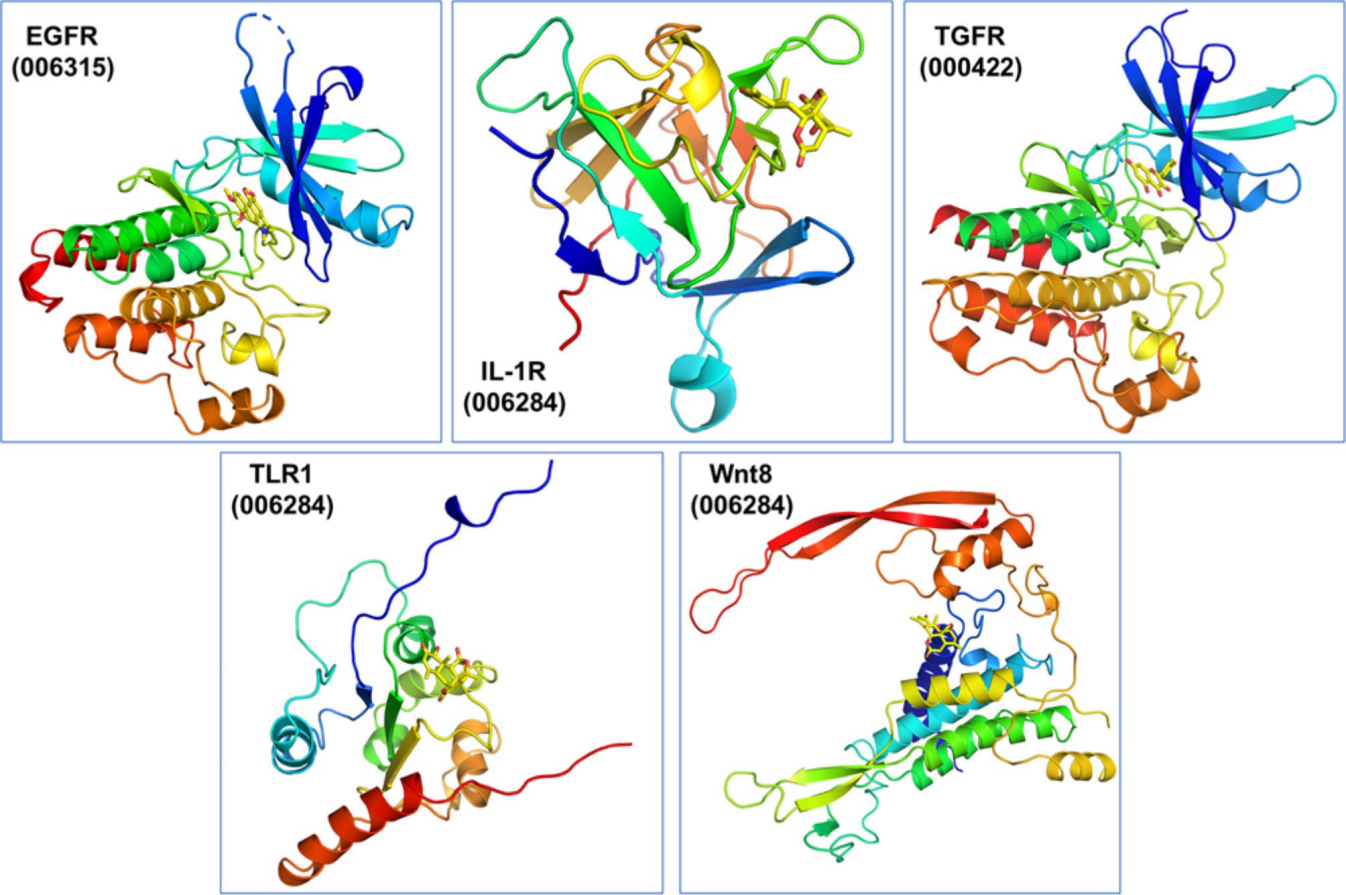
Binding pattern of the highest scoring compounds to human EGFR, IL-1R, TGFR, TLR, and Wnt8 proteins. The colored curly structure represents the amino acid spatial sequence of the target protein, and the yellow structure in the protein cavity represents small molecules. The free energies of binding to the active cavities of human EGFR, IL-1R, TGFR, TLR1 and Wnt proteins of the highest scoring compounds dehydrocrebanine (006315), ailanthone (006284), kaempferol (000422), ailanthone (006284), and ailanthone (006284) are − 8.7, − 8.0, − 9.2, − 7.7, and − 8.5 kcal/mol, respectively

Figure 1 shows that the compounds bind closely to the surface of human IL-1R, TLR, EGFR, TGFR, and Wnt proteins, and there is a good spatial match between them. All of the compounds bind to the active space of the corresponding protein.

### Microscopic binding of the highest scoring compound to amino acid residues near the active site of the target protein

**Fig. 2:**
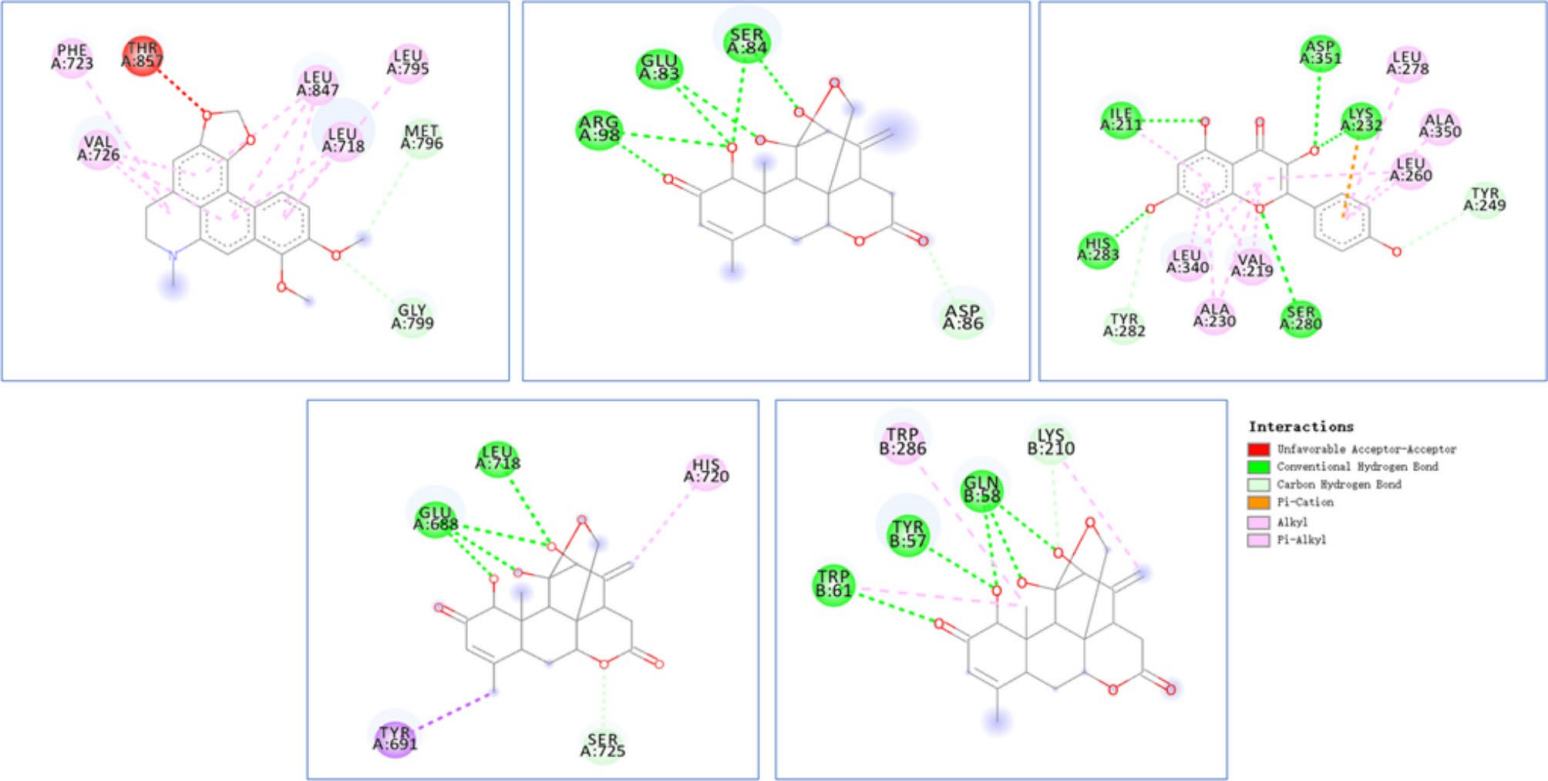
Microscopic binding of the highest scoring compound to amino acid residues near the active site of the target protein. Green circles and lines represent conventional hydrogen bonds; light blue circles and lines represent carbon hydrocarbon bonds; orange circles and lines represent π–cation interactions; pink circles and lines represent alkyls; and light pink circles and lines represent π–alkyl interactions. The letter and the number under the amino acid represent the abbreviated name and the primary structure of theamino acid sequence

Figure 2 shows that dehydrocrebanine has a noncovalent bond with the residues between the amino acid motifs of threonine (THR) 857 near the active site of human EGFR protein, and ailanthone interacts with the ASP active site of human IL-1R protein. Partic acid (86 amino acid residues) has hydrogen-bonding interactions. Kaempferol forms a cation–π interaction with lysine (LYS) 232 at the active site of human TGFR protein, and ailanthone forms a π–alkyl interaction with tryptophan (TYR) 691 near the active site of TLR. Ailanthone forms a π–alkyl interaction with the LYS 210 active site of human Wnt protein, forming hydrogen-bond interactions.

### Inhibitory effect of ailanthone on cell proliferatio

**Fig. 3:**
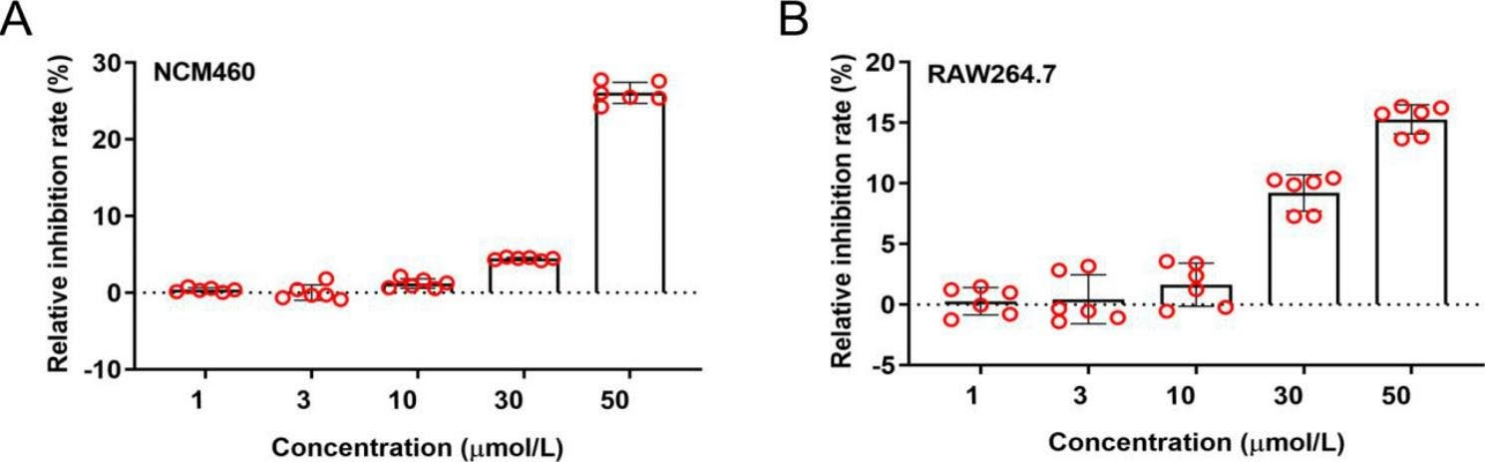
Inhibitory effect of ailanthone on cell proliferation. **(A)** The relative inhibition rate of ailanthone was tested on NCM460 cells by CCK-8 experiment. **(B)** The relative inhibition of ailanthone was tested on RAW264.7 cells by CCK-8 experiment. N = 6

Ailanthone was found to be able to combine with L-1R, TLR1, and Wnt at the same time through virtual screening of front molecule docking. It is worth noting that the docking scores were high. However, the computer results could not replace the experiment. The cytotoxicity of ailanthone was tested on cells by CCK-8 experiment (NCM460; RAW264.7). The results showed that cell proliferation was not significantly affected at the concentrations of 1, 3, and 10 µmol of ailanthone (Fig. 3). This safe dose can be used to observe the anti-inflammatory activity of ailanthone.

### Ailanthone can inhibit the activity of TNF-α, IL-6, and IL-1β

**Fig. 4:**
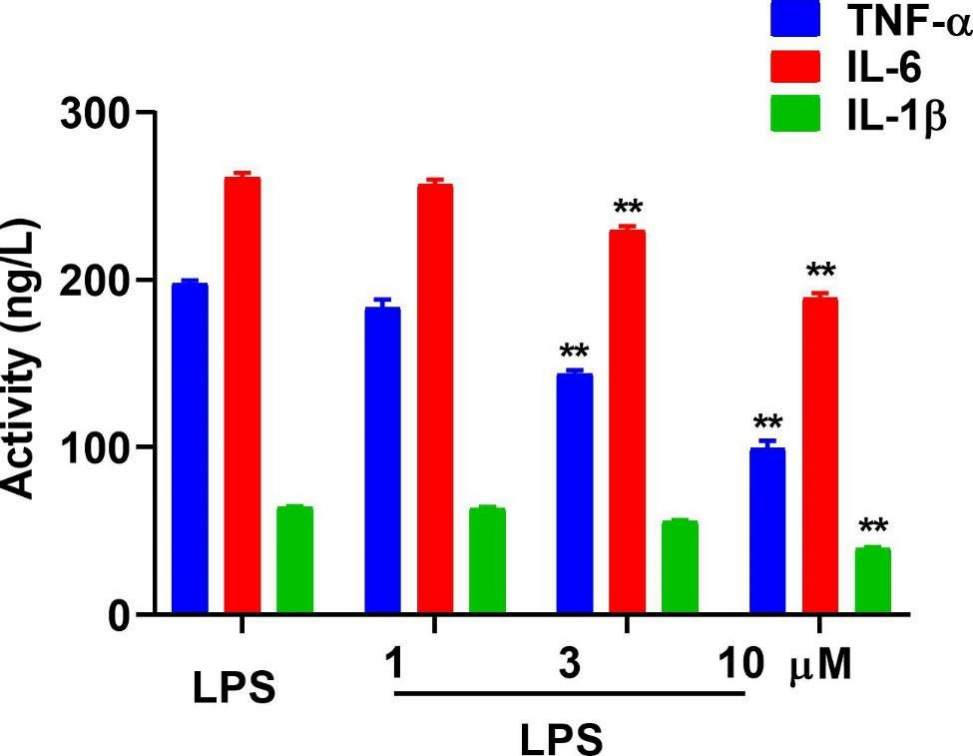
Ailanthone can inhibit the activity of TNF-α, IL-6, and IL-1β. The activity of the pro-inflammatory cytokines was observed in an ELISA experiment on RAW264.7 cells. N = 6. **P < 0.01

The activity of TNF-α, IL-6, and IL-1β is closely related to the development of UC. The concentrations of 1, 3, and 10 µM of ailanthone were used to observe the effect on the activity of TNF-α, IL-6, and IL-1β in RAW264.7 cells (Fig. 4). The levels of these pro-inflammatory cytokines decreased after administration of ailanthone (10 µM).

## Discussion

In recent years, there have been many studies on the treatment of UC with TCM [37]. Many components in TCM, such as *Patrinia scabiosaefolia Fisch* [38], *Lindera aggregata (Sims) Kosterm* [39], *Glycyrrhizae* [40], *Angelica sinensis* [41], and BAA [13], have been reported to have therapeutic effects. Particularly significant among these components are flavonoids, glycosides, phenols, polysaccharides, and alkaloids. BAA, a common herbal medicine used in the TCM treatment of UC, has attracted increasing attention. Modern medical research has shown that BAA and its preparations have a good curative effect on UC [13]. At present, research on the treatment of UC is in the initial stage, and its pharmacodynamic substances have not been fully investigated [42]. The molecular docking virtual screening technology used to screen the pharmacodynamic substances (active component groups) of BAA in the treatment of UC is relatively simple and can obtain more accurate results.

Molecular docking technology docks known small-molecule active substances with related target proteins, which can clarify the mechanism of action between active TCM ingredients and targets at the molecular level [43, 44]. At present, molecular docking technology plays an important role in elucidating the mechanism of action between active ingredients of TCM [45, 46] and related target proteins in many diseases, such as cardiovascular disease [25] and cancer [26]. Therefore, we conducted preliminary screening with reference to the five principles of drug-like standards and other screening conditions of “drug similarity” and obtained 22 compounds. The 22 compounds obtained from the secondary screening were molecularly docked with human IL-1R, TLR, EGFR, TGFR, and Wnt proteins using AutoDock Vina software, and the binding conformation was scored using a scoring function. The free energies of the highest scoring compounds binding to the active cavity of human IL-1R, TLR, EGFR, TGFR, and Wnt proteins were − 8.7, − 8.0, − 9.2, − 7.7, and − 8.5 kcal/mol, respectively. It is generally believed that when the absolute value is greater than 7 kcal/mol, the possibility of the combination of the compound and the protein is high [47]. The larger the absolute value of the binding constant, the lower the free energy required for the compound to bind [48]. The compound with the highest function score can be selected in order to construct a compound–protein binding diagram, which can visually convey the binding mode of the active compound with human IL-1R, TLR, EGFR, TGFR, and Wnt proteins and their interaction with the surrounding amino acid residues. It is worth noting that ailanthone was found to be able to combine with L-1R, TLR1, and Wnt proteins at the same time through virtual screening of front molecule docking, and the binding score was very high. This compound was further tested in vitro. Ailanthone had no significant inhibitory effect on cell proliferation within the concentration range of 1–10 µM, and a 10-µM concentration significantly inhibited the pro-inflammatory response induced by LPSa.

## Conclusions

Briefly, in this study, the effective substances of BAA in treating UC were studied based on Virtual Screening–Molecular Docking–Activity Evaluation technology. We found that ailanthone had good activity in vitro. Although further animal experiments are needed for verification, ailanthone has a high possibility of becoming an effective drug in the future.

## Data Availability

The data used to support the findings of this study are available from the corresponding author upon reasonable request.

## Abbreviations

BAA: Bark of *Ailanthus altissima* (Mill.) swingle
DMSO: Dimethyl sulfoxide
EGFR: Epidermal growth factor receptor
IL-1β: Interleukin-1β
IL-1R: Interleukin-1 receptor
IL-6: Interleukin-6
IC_50_: Half maximal inhibitory concentration
LPS: Lipopolysaccharide
NO: Nitric oxide
NOS: Nitric oxide synthase
PDB: Protein data bank
TCM: Traditional Chinese medicine
TCMSP: Traditional Chinese Medicine Systems Pharmacology
TGFR: Transforming growth factor receptor
TLR: Toll-like receptor
TNF-α: Tumor necrosis factor-α
UC: Ulcerative colitis
